# Evolution of publicly available large language models for complex decision-making in breast cancer care

**DOI:** 10.1007/s00404-024-07565-4

**Published:** 2024-05-29

**Authors:** Sebastian Griewing, Johannes Knitza, Jelena Boekhoff, Christoph Hillen, Fabian Lechner, Uwe Wagner, Markus Wallwiener, Sebastian Kuhn

**Affiliations:** 1 Institute for Digital Medicine, Philipps-University Marburg, Marburg, Germany; 2 Institute for Artificial Intelligence in Medicine, Philipps-University Marburg, Marburg, Germany; 3 Department of Gynecology and Obstetrics, Philipps-University Marburg, Marburg, Germany; 4 Department of Gynecology and Obstetrics, Martin-Luther University Halle-Wittenberg, Halle, Germany; 5 Department of Gynecology and Gynecologic Oncology, University Medical Center Hamburg-Eppendorf, Hamburg, Germany; 6 Kommission Digitale Medizin, Deutsche Gesellschaft für Gynäkologie und Geburtshilfe, Berlin, Germany

**Keywords:** Large language models, Breast cancer, Artificial intelligence, ChatGPT, Tumor board

## Abstract

**Purpose:**

This study investigated the concordance of five different publicly available Large Language Models (LLM) with the recommendations of a multidisciplinary tumor board regarding treatment recommendations for complex breast cancer patient profiles.

**Methods:**

Five LLM, including three versions of ChatGPT (version 4 and 3.5, with data access until September 3021 and January 2022), Llama2, and Bard were prompted to produce treatment recommendations for 20 complex breast cancer patient profiles. LLM recommendations were compared to the recommendations of a multidisciplinary tumor board (gold standard), including surgical, endocrine and systemic treatment, radiotherapy, and genetic testing therapy options.

**Results:**

GPT4 demonstrated the highest concordance (70.6%) for invasive breast cancer patient profiles, followed by GPT3.5 September 2021 (58.8%), GPT3.5 January 2022 (41.2%), Llama2 (35.3%) and Bard (23.5%). Including precancerous lesions of ductal carcinoma in situ, the identical ranking was reached with lower overall concordance for each LLM (GPT4 60.0%, GPT3.5 September 2021 50.0%, GPT3.5 January 2022 35.0%, Llama2 30.0%, Bard 20.0%). GPT4 achieved full concordance (100%) for radiotherapy. Lowest alignment was reached in recommending genetic testing, demonstrating a varying concordance (55.0% for GPT3.5 January 2022, Llama2 and Bard up to 85.0% for GPT4).

**Conclusion:**

This early feasibility study is the first to compare different LLM in breast cancer care with regard to changes in accuracy over time, i.e., with access to more data or through technological upgrades. Methodological advancement, i.e., the optimization of prompting techniques, and technological development, i.e., enabling data input control and secure data processing, are necessary in the preparation of large-scale and multicenter studies to provide evidence on their safe and reliable clinical application. At present, safe and evidenced use of LLM in clinical breast cancer care is not yet feasible.

## What does this study add to the clinical work?

**Table:** 

This early feasibility study demonstrates that publicly available Large Language Models (LLM), especially GPT4, increasingly align with the decision-making of a multidisciplinary tumor board regarding high complexity breast cancer treatment choices. It indicates that methodological advancement, i.e. the optimization of prompting techniques, and technological development, i.e. enabling data input control and secure data processing, are necessary in the preparation of large-scale and multicenter studies. At present, safe and evidenced use of LLM in clinical breast cancer care is not yet feasible.	

## Introduction

In Germany, invasive breast cancer is the most prevalent cancer affecting women, with the annual national incidence exceeding 70,000 cases [1]. The implementation of a comprehensive nationwide mammography screening program between the years of 2005 to 2009 resulted in an initial peak in detected breast cancer cases. Subsequently, the increased efforts led to a consistent reduction in the incidence of advanced tumors and a gradual decline of primary disease. Despite these improvements, a high disease burden of breast cancer persists and given the aging demographic, a future increase in the incidence of breast cancer is anticipated. This development is accompanied by intensified shortage in healthcare professionals and care capacity [1, 2]. In addition, extensive research continuously expands the spectrum of treatment modalities, encompassing surgical interventions, endocrine therapy, radiotherapy, both neoadjuvant and adjuvant chemotherapy, and genetic testing for hereditary breast and ovarian cancer syndromes [3]. Moreover, the swift advancement in diagnostic and treatment technologies, including increasing adoption of next-generation sequencing, genetic arrays for the prediction of disease prognosis or chemotherapy benefit and the use of precision-targeted therapies such as antibody-drug conjugates, shape a transformative phase in gynecological oncology [4, 5]. This development is marked by abundance of evidenced knowledge and health data, which increasingly overwhelm practitioners in terms of complexity [6]. There is a growing optimism that technological innovations will bridge the gap between scientific possibilities and practical healthcare delivery by providing support to caregivers and will enable more individualized and effective treatment strategies in an environment with high volumes of data [7].

High expectations are set on artificial intelligence-based clinical decision support tools to augment doctoral intelligence in order to keep pace with this rapid development [8, 9]. Historically, the cumbersome digitization of German healthcare has led to a gap between technological capabilities and current practices, which keeps on widening [10]. A nationwide survey conducted by the Commission Digital Medicine of the German Association of Gynecology and Obstetrics (DGGG) revealed high heterogeneity in digital infrastructure within the field of gynecology, characterized by low interoperability and outdated systems, leading to dissatisfaction among healthcare providers [11]. In contrast, most gynecology specialists are optimistic that digitization could ease their growing workloads, enhance patient care, and foresee the adoption of smart algorithms to assist in patient treatment [12]. In the meantime, it has become a normality on the patient’s side to assess new symptoms digitally before visiting a doctor, i.e., using online-search engines and dedicated app-based symptom checkers [13–15]. This includes the recent widespread availability of Large Language Models (LLM), with tech-savvy individuals increasingly turning to public chatbots for health-related inquiries [9, 16]. This shift toward relying on easily accessible online resources, evolving from simple Google keyword searches to consulting advanced tools like ChatGPT, highlights a new reality that likewise demands to promote scientific evidence in the medical use of LLM.

The emergence of publicly available LLM in artificial intelligence has opened a new field in medical research, which still lacks the definition of methodological guard rails and best practices. Preliminary proof-of-concept analyses have indicated potential in using these models as supplementary tools in tumor boards [16–21]. In breast cancer care, few preliminary assessments have explored the accuracy of LLM in supporting decision-making through the evaluation of brief clinical scenarios [22, 23], but also high complexity cases [24]. The most recent literature increasingly challenges the consistency of LLM, highlighting significant changes in explanatory value over short intervals while emphasizing the necessity for their ongoing monitoring [25]. A scientific discussion has initiated on whether LLM will facilitate the implementation of increasingly complex evidence-based treatment guidelines in clinical routine or may serve as a possible guideline navigator for the professional user [16–21]. Furthermore, the question remains as to how to direct the technological and methodological development of LLM before initiating larger preclinical and clinical trials to generate further evidence on the technology’s application in breast cancer care.

To date, there is no literature in breast cancer care that compares different LLM and considers their monitoring with regard to changes in accuracy over time, i.e., with access to more data or through technological upgrades. Therefore, this early feasibility study investigated five different versions of publicly available LLM regarding their concordance of recommendations for complex breast cancer case examples at different stages of development and points in time. Based on its findings, it aims to conclude on how to direct further development and the scientific approach to LLM in breast cancer care.

## Methods

### Patient profiles

Following the breast cancer guidelines of the German Association of Gynecology and Obstetrics (DGGG) (version 4.4, May 2021, AWMF-registration number 032/0456OL), 20 patient profiles (P1-20) were designed to reflect the patho- and immunomorphological variety of breast cancer in comprehensive and structured manner (Tables 1 and 2) [24]. The use of publicly available LLM is limited to fictitious profiles at the current state, as data processing via international servers does not ensure data integrity in accordance with European (General Data Protection Regulation, GDPR) or German data protection standards (Datenschutz-Grundverordnung, DSVGO). This limits the current exploration of LLM to a preclinical simulation environment. Since no patient-related data was used, an ethics vote was waived by the Research Ethics Committee of Philipps-University Marburg (23-300 ANZ).

**Table 1:** Patient profiles 1–10 [24]

P1–10	P1	P2	P3	P4	P5	P6	P7	P8	P9	P10
Patient profiles	Postmenopausal luminal A N−	Postmenopausal luminal A N+	Premenopausal luminal A N−	Premenopausal luminal A N+	Postmenopausal luminal B Her2− N−	Postmenopausal luminal B Her2− N+	Premenopausal luminal B Her2− N−	Premenopausal luminal B Her2+ N+	Postmenopausal Her2+ER/PR- N−	Postmenopausal Her2+ER/PR- N+
Age	62	61	50	45	62	58	40	35	58	65
Menopause status	Post	Post	Post	Post	Post	Post	Post	Post	Post	Post
ECOG	0	0	1	1	0	1	0	0	1	2
Previous illness	Bronchial asthma (no long-term therapy, acute therapy with inhaled corticosteroids and formoterol), arterial hypertension (with antihypertensive triple combination of diuretic, calcium antagonist and AT II antagonist)	Hypothyroidism (with L-thyroxine medication)	Relapsing remitting multiple sclerosis (last episode 5 years ago, no long-term medication)	HELLP Syndrome at first pregnancy at age of 34	Diabetes mellitus type 1, arterial hypertension (with ACE inhibitor medication), hemorrhoids	Crohn’s disease (with continuous therapy with TNF-alpha inhibitors)	Deep vein thrombosis at age 25 while on contraceptive medication, heterozygous factor V Leiden	Colitis ulcerosa, Hashimoto-thyroiditis (with L-thyroxine medication)	COPD GOLD B (with inhaled long-acting muscarinic receptor antagonists and inhaled long-acting ß2 sympathomimetics medication)	Atrial fibrillation (with direct oral anticoagulant and beta-blocker medication), pulmonary artery embolism at the age of 65 following immobilization during right-sided total hip arthroplasty
Previous surgical treatment	Transverse laparotomy for hysterectomy because of hypermenorrhea and uterine myomatosus at age of 42, laparoscopic cholecystectomy at the age of 45, open appendectomy at the age of 29	Open cholecystectomy at the age of 35, breast-conserving tumorectomy for right-sided fibroadenoma at the age of 32, uterine curettage after early abortion at age of 20	Tonsillectomy in childhood, open appendectomy for complicated appendicitis without free perforation at the age of 27	Postpartum cardiomyopathy with intensive care ECMO support, Roux-Y gastric bypass for obesity (BMI 50) at the age of 32	Mamma abscess cleavage on the right side at the age of 35, open hemorrhoidectomy according to Milligan-Morgan at the age of 40	Bowel-sparing resection for ileum stenosis at the age of 35, open appendectomy at the age of 25, longitudinal laparotomy for mechanical ileus at the age of 55	Open appendectomy at the age of 28	Laparoscopy for cyst extirpation of left ovarian cyst at age 30	none	Right-sided total hip arthroplasty at the age of 65
Birth history	1 vaginal birth at age of 32, 1 cesarean at the age of 34, 1 early abortion at the age of 30	4 vaginal births at the age of 25, 27, 29 and 30, 1 early abortion at the age of 20	no prior birth	2 cesareans at the age of 34 and 38	4 vaginal births at the age of 18, 20, 28 and 30	no prior birth	1 vaginal birth at the age of 39	no prior birth	2 vaginal births at the age of 28 and 30	2 vaginal births at the age of 23 and 30 and 1 cesarean at the age of 35
Oncologic family history	Maternal aunt with colon cancer at the age of 62	Maternal female cousin with hodgkin lymphoma at the age of 30	no prior oncologic family history	Paternal uncle with prostate cancer at the age of 65	Paternal uncle with colon cancer at the age of 40, paternal grandfather with colon cancer at the age of 60, paternal cousin with colon cancer at the age of 35	Maternal grandmother with breast cancer at the age of 80	Sister-in-law with breast cancer at the age of 30	Paternal grandmother with breast cancer at the age of 70, paternal aunt with breast cancer at the age of 50, maternal uncle with pancreatic cancer at the age of 60	Maternal grandmother with endometrial cancer at the age of 75, mother with bile duct carcinoma at the age of 60	Sister with childhood acute lymphoblastic leukemia, father with gastric carcinoma at the age of 50
Previous surgical treatment	BCT+SLN right	BCT+SLN left	BCT+SLN right	BCT+SLN left	BCT+SLN right	BCT+SLN left	BCT+SLN left	BCT+SLN right	BCT+SLN left	MT+SLN right
TNM	pT1bN0MX	pT2(2)pN1aM0	pT1apN0MX	pT1cpN1aM0	pT3pN0M0	pT3(3)pN1aM0	pT2pN0M0	pT2pN1cM0	pT1apN0M0	pT3pN1aM0
Resection margin	R0, 5 mm	R0, 6 mm	R0, 1 mm	R1 on lateral aspect	R0, 0.1 mm	R0, 7 mm	R1 on lateral aspect	R0, 2 mm	R0, 0.05 mm	R0, 10 mm
Histologic subtype	NST	Invasive-lobular	Mucinous	NST	Invasive-lobular	NST	Tubular	Invasive-lobular	NST	NST
Grading	G1	G2	G1	G2	G1	G2	G2	G3	G2	G2
UL/BL	Unilateral	Unilateral	Unilateral	Unilateral	Unilateral	Unilateral	Unilateral	Unilateral	Unilateral	Unilateral
MF/MC	Monofocal and -centric	Monocentric and multifocal, 2 foci	Monofocal and -centric	Monofocal and -centric	Monofocal and -centric	Monocentric and multifocal, 3 foci	Monofocal and -centric	Monofocal and -centric	Monofocal and -centric	Monofocal and -centric
ER	95%	85%	95%	100%	80%	75%	90%	75%	5%	0%
PR	80%	80%	90%	100%	75%	90%	50%	75%	1%	0%
Her2	Negative (IHC 0)	Negative (IHC 1+)	Negative (IHC 0)	Negative (IHC 0)	Negative (IHC 1+)	Negative (IHC 0)	Negative (IHC 2+, ISH negative)	Positive (IHC 3+)	Positive (ISH positive)	Positive (ICH 3+)
Ki-67	10%	15%	8%	10%	35%	28%	30%	40%	20%	35%

*N* ± nodal positive or negative, *Her2* ± Her2 positive or negative, *BCT* breast-conserving tumorectomy, *SLN* sentinellymphnodectomy, *MT* mastectomy, *UL/BL* uni- versus bilaterality, *MF/MC* multifocality or -centricity, *ER* estrogen receptor, *PR* progesterone receptor, *Her2* Her2 status, *Ki-67* Ki-67-proliferation-index

**Table 2:** Patient profiles 11–20 [24]

P11–20	P11	P12	P13	P14	P15	P16	P17	P18	P19	P20
Patient profiles	Premenopausal Her2+ER/PR- N−	Premenopausal Her2+ER/PR- N+	Postmenopausal triple Negative N−	Postmenopausal triple Negative N+	Premenopausal triple Negative N−	Premenopausal triple Negative N+	Postmenopausal DCIS, clear resection margin	Premenopausal DCIS, clear resection margin	Postmenopausal DCIS, narrow resection margin	Inflammatory Breast cancer
Age	32	42	56	65	29	35	70	38	72	36
Menopause status	pre	pre	post	post	pre	pre	post	pre	post	pre
ECOG	0	0	1	1	0	0	2	0	1	0
Previous illness	Insulin-dependent gestational diabetes during the first pregnancy at the age of 20, postpartum depression at the age of 20	Pulmonary artery embolism after pelvic vein thrombosis at the age of 26, antiphospholipid syndrome with anti-cardiolipin antibodies (with permanent oral anticoagulation with phenprocoumon)	Insulin-dependent diabetes mellitus type 2, obesity with BMI of 43, obstructive sleep apnea syndrome, secondary arterial hypertension (with ACE inhibitor medication)	Addison’s disease (currently under hydro- and fludrocortisone medication)	none	Paranoid schizophrenia (under stable condition with current olanzapine medication)	Arterial hypertension (with with AT II antagonist medication)	AV-node re-entry tachycardia (with beta-blocker medication)	Chronic lymphocytic leukemia Stadium A	None
Previous surgical treatment	None	None	Abdominoplasty at the age of 50, bilateral mammary reduction mammoplasty for mammary hypertrophy at the age of 35	Total knee replacement on the left side at the age of 50	None	None	Vaginal hysterectomy with bilateral adnexectomy for uterine prolapse at the age of 55, transcatheter aortic valve implantation due to aortic valve stenosis at the age of 69	None	Total shoulder arthroplasty on the left side	None
Birth history	1 vaginal birth at the age of 20	6 early abortions between the age of 20 and 26	3 vaginal births at the age of 20, 21 and 25	3 cesareans at the age of 25, 28 and 35	No prior birth	No prior birth	2 vaginal births at the age of 16 and 20	1 cesarean at the age of 36	1 vaginal birth at the age of 22	No prior birth
Oncologic family history	Father with bronchial carcinoma at the age of 60, sister with osteosarcoma at the age of 18	Father with colon cancer at the age of 45, paternal grandmother with endometrial cancer at the age of 65, paternal uncle with urothelial carcinoma of the renal pelvis at the age of 55	Mother with breast cancer at the age of 40	Paternal grandmother with pancreatic cancer at the age of 59, maternal aunt with colon cancer at the age of 60	Mother with breast cancer at the age of 65, maternal grandmother with breast cancer at the age of 70	Maternal grandmother with endometrial cancer at age of 60, paternal uncle with rectum carcinoma at the age of 50	1 sister with peritoneal cancer at the age of 60, maternal grandmother with ovarian cancer at the age of 65	Paternal grandfather with prostate cancer at the age of 65, mother with chronic myeloid leukemia at the age of 70	Father with colon cancer at age 55	No cancer history
Previous surgical treatment	BCT+SLN left	BCT+SLN right	BCT+SLN left	None so far	None so far	None so far	MT right	BCT left and right	BCT+SLN left	None so far
TNM	pT2pN0M0	pT2pN1M0	pT1apN0M0	cT3pN+pM1 (OSS)	cT2cN0M0 on left side and cT1bcN0M0 on right side	cT2pN+pM1 (HEP)	pTis (size of the lesion 4.3 cm)	pTis on left and right side (size of the lesions: 2.3 cm on left and 3.2 cm on right side)	pTis (size of lesion 1.5 cm)	cT4dpN+M0
Resection margin	R0, 4 mm	R0, 2 mm	R0, 1 mm	Not applicable	Not applicable	Not applicable	R0, 10 mm	R0, 4 mm on left, 5 mm on right side	R0, 0.01 mm	Not applicable
Histologic subtype	NST	NST	NST	NST	NST on left and right side	NST	Not applicable	Not applicable	Not applicable	NST, inflammatory breast cancer with lymphangiosis carcinomatosa
Grading	G2	G3	G2	G3	G3	G3	Not applicable	Not applicable	Not applicable	G3
UL/BL	Unilateral	Unilateral	Unilateral	Unilateral	Bilateral	Unilateral	Unilateral	Bilateral	Unilateral	Unilateral
MF/MC	Monofocal and -centric	Monofocal and -centric	Monofocal and -centric	Not applicable	Not applicable	Not applicable	Monofocal and multi-centric, 2 centers	Monofocal and -centric on left and right side	Monofocal and -centric	Not applicable
ER	0%	0%	0%	0%	0% on left and right side	1%	95%	100% on left and right side	100%	5%
PR	0%	5%	0%	0%	0% on left and right side	2%	90%	100% on left and right side	100%	5%
Her2	Positive (ICH 3+)	Positive (ISH positive)	Negative (IHC 1+)	Negative (IHC 0)	Negative (IHC 0) on left and right side	Negative (IHC 1+)	Not applicable	Not applicable	Not applicable	Positive (ISH positive)
Ki-67	65%	80%	40%	60%	70% left, 85% on right	80%	Not applicable	Not applicable	Not applicable	70%

*N* ± nodal positive or negative, *Her2* ± Her2 positive or negative, *BCT* breast-conserving tumorectomy, *SLN* sentinellymphnodectomy, *MT* mastectomy, *UL/BL* uni- versus bilaterality, *MF/MC* multifocality or -centricity, *ER* estrogen receptor, *PR* progesterone receptor, *Her2* Her2 status, *Ki-67* Ki-67-proliferation-index

### Prompting model

Prompting was carried out using a previously used, standardized input model for high complexity clinical cases (supplementary file 1) [24]. Prompts had to be slightly adjusted for patient profiles without previous surgical intervention (P14-16, P20) and ductal carcinoma in situ (DCIS) (P17-19).

### Large language model selection

Five different LLM were utilized for comparison. GPT (ChatGPT Generative Pre-trained Transformer; by OpenAI LP, San Francisco, California, USA) was analyzed in three different development versions (GPT3.5 version September 2021, GPT3.5 version January 2022, GPT4 version April 2023) to trace the evolution over time and with access to more data or through technological upgrade. Besides, the selection of Llama2 70bn (version December 2022; Large Language Model Meta AI 2 70 billion parameters; by Meta, Menlo Park, California, USA) and Bard (version January 2023; by Google LLC, Mountain View, California, USA) enabled the comparison of two further commonly used LLMs.

### Model execution

On July 21, 2023, the high complexity cases were presented in a randomized and blinded order to the multidisciplinary tumor board (MTB) of the partnering accredited gynecologic oncology center (supplementary file 1). On the same date, prompting was carried out in GPT3.5 version September 2021. GPT3.5 version January 2022, Llama2, Bard and GPT4 (version April 2023) were queried on December 6, 2023 (supplementary file 2).

### Comparative assessment

Different treatment modality recommendations were assessed: surgical treatment (ST), endocrine treatment (ET), systemic treatment or chemotherapy (CT), radiotherapy (RT) and genetic testing (GT). The determination of treatment was recorded on a binary scale for each modality (recommended versus not recommended). Since the initially chosen prompting model did not include a query of multi-gene assays for the prediction of disease prognosis or chemotherapy benefit, the LLM did not provide an answer in this regard. Hence, profiles that were advised by the MTB to undergo the respective tests were excluded from analysis. As LLM depend on effective prompting, the suggested treatment options were categorized as recommended treatments (see supplementary file 1 and 2). Concordance between LLM and MTB treatment suggestions was assessed using descriptive statistics for each individual patient profile and specific treatment option.

## Results

### Comparative assessment per patient profile

**Fig. 1:**
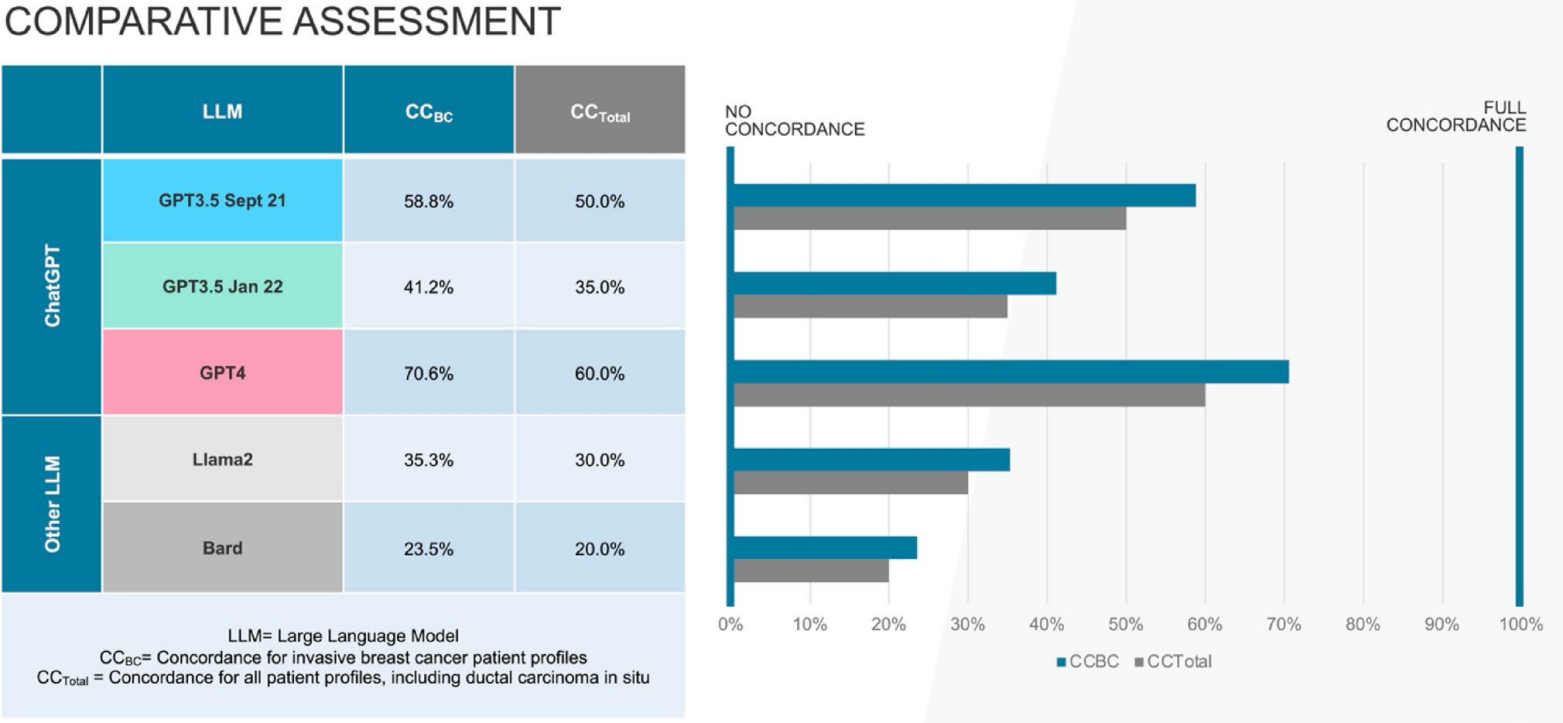
Comparison of average performance according to type of LLM

Overall concordance between LLM and MTB recommendations was highest for GPT4 with 12/20 (60.0%), followed by GPT3.5 version September 21 (50.0%; 10/20) and GPT3.5 version January 22 (35.0%; 7/20) (see Table 3). For invasive breast cancer patients exclusively (CC_BC_), GPT4´s concordance amounts to 70.6% (12/17). Removing GT from assessment provides full concordance for invasive breast cancer of 82.4% for GPT4 and GPT3.5 version September 2021 (14/17). P7 had to be excluded from the partial evaluation as MTB recommended to perform a genetic array using Endopredict® (Myriad Genetics GmbH, Zurich, Switzerland) to assess the need for chemotherapy for the specific patient profile (see Fig. 1).

**Table 3:** Concordance according to patient profile per LLM

Overall concordance per patient profile per LLM						
Patient profiles		ChatGPT versions			Other LLM	
		GPT3.5 Sept 21	GPT3.5 Jan 22	GPT4	Llama2	Bard
Postmenopausal luminal A N−	1	No	No	Yes	No	No
Postmenopausal luminal A N+	2	No	No	No	No	No
Premenopausal luminal A N−	3	Yes	Yes	Yes	No	No
Premenopausal luminal A N+	4	Yes	No	No	No	No
Postmenopausal luminal B Her2− N−	5	Yes	No	No	No	No
Postmenopausal luminal B Her2− N+	6	No	No	Yes	No	Yes
Premenopausal luminal B Her2− N−	7	No	No	No	No	Yes
Premenopausal luminal B Her2+N+	8	Yes	Yes	Yes	Yes	No
Postmenopausal Her2+ER/PR- N−	9	No	No	Yes	No	Yes
Postmenopausal Her2+ER/PR- N+	10	No	No	No	No	Yes
Premenopausal Her2+ER/PR- N−	11	Yes	Yes	Yes	Yes	No
Premenopausal Her2+ER/PR- N+	12	Yes	Yes	Yes	Yes	No
Postmenopausal triple negative N−	13	Yes	Yes	Yes	Yes	No
Postmenopausal triple negative N+	14	Yes	Yes	Yes	No	No
Premenopausal triple negative N−	15	Yes	Yes	Yes	Yes	No
Premenopausal triple negative N+	16	No	No	Yes	Yes	No
Postmenopausal DCIS, clear resection margin	17	No	No	No	No	No
Premenopausal DCIS, clear resection margin	18	No	No	No	No	No
Postmenopausal DCIS, narrow resection margin	19	No	No	No	No	No
Inflammatory breast cancer	20	Yes	No	Yes	No	No
		**50.0%**	**35.0%**	**60.0%**	**30.0%**	**20.0%**

*LLM* large language model, *PP* patient profile, *N*+ nodal positive, *N−* nodal negative, *Her2*+ Her2 positive, *Her2−* Her2 negative, *DCIS* ductal carcinoma in situ, *Sept* September, *Jan* January

### Comparative assessment according to treatment option

**Fig. 2:**
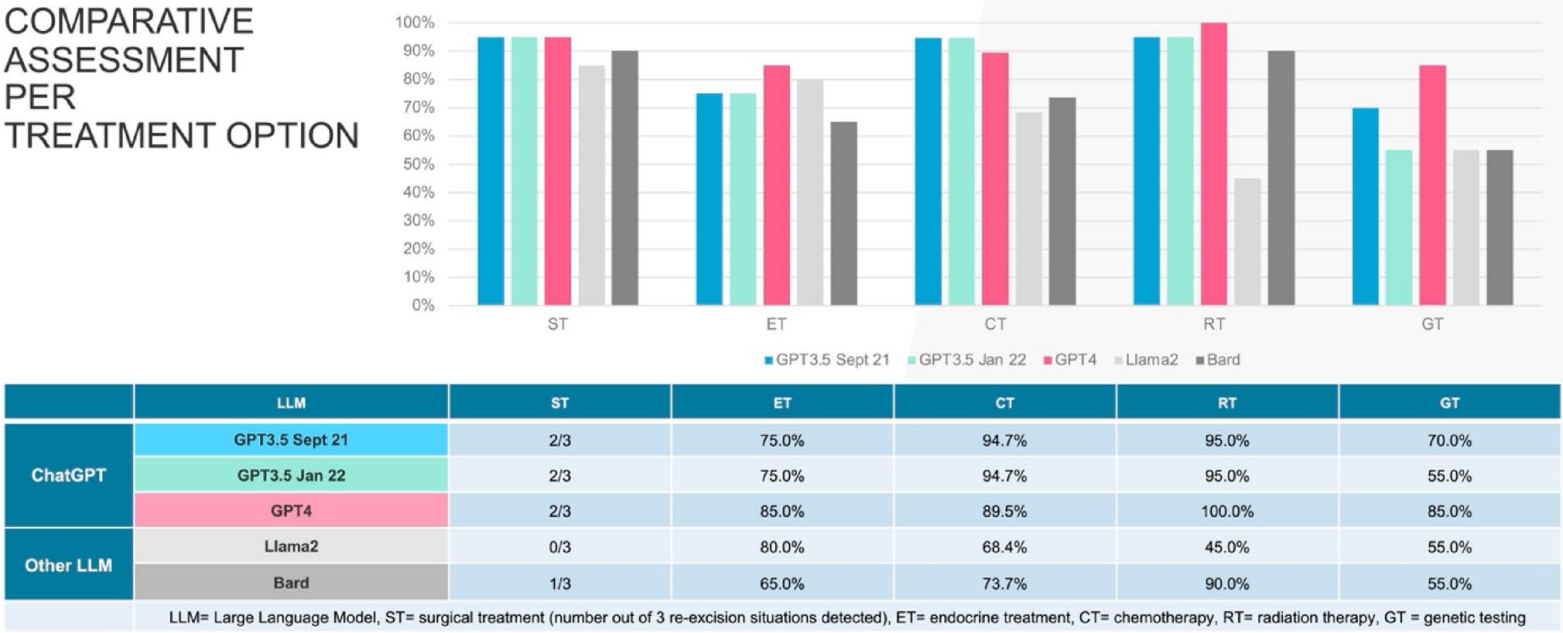
Comparative assessment according to type of LLM and treatment option

GPT4 achieved full concordance for RT (100%; 20/20) and the highest concordance for ET and GT by 85% (each 17/20). Regarding CT, GPT3.5 scored highest with 94.7% (18/19) followed by GPT4 with 89.5% (17/19) (see Fig. 2).

### Longitudinal assessment of GPT versions

**Fig. 3:**
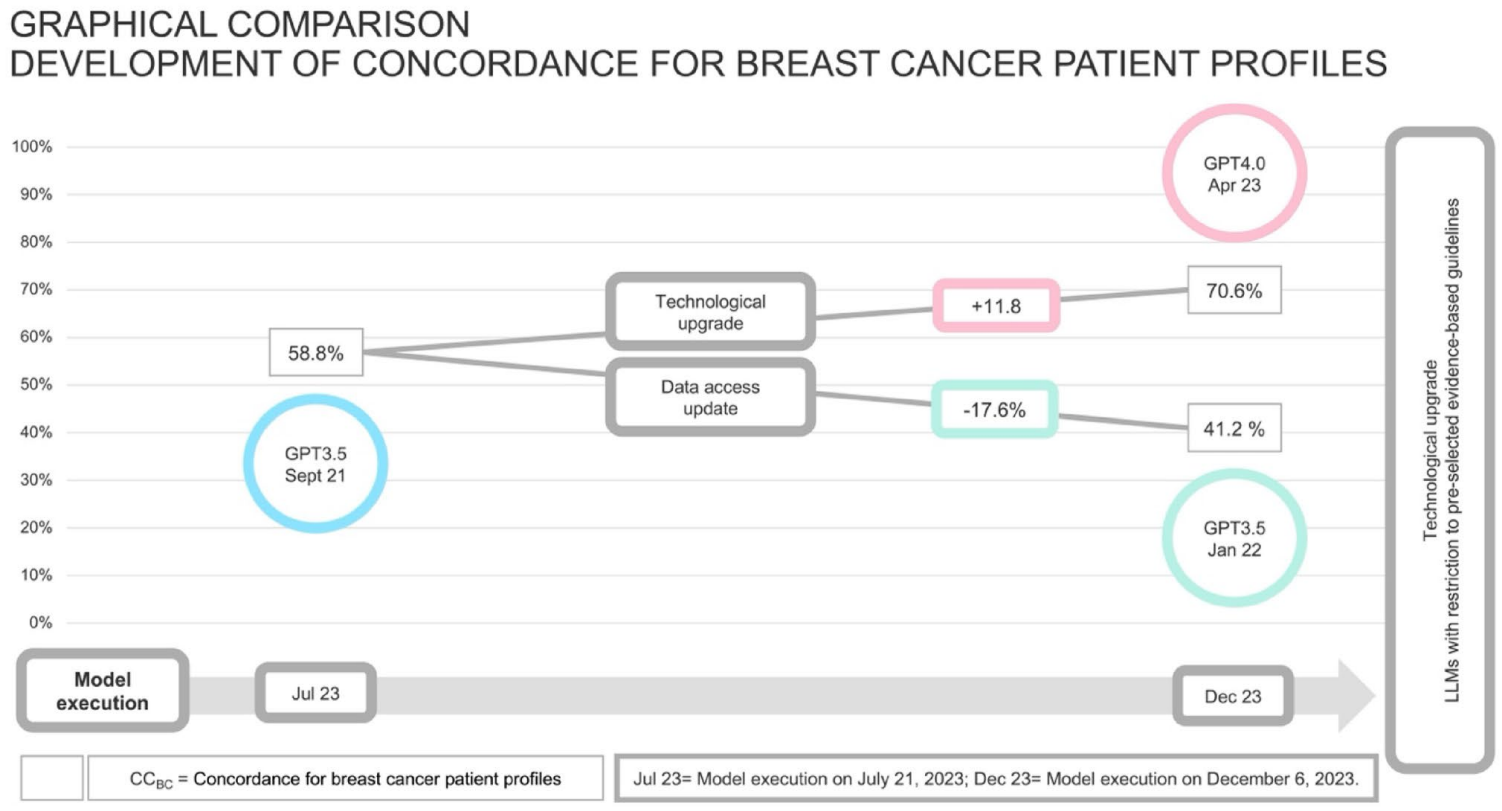
Development of concordance for breast cancer patient profiles for GPT versions

Figure 3 demonstrates the alternating accuracy of GPT versions regarding the concordance on breast cancer patient profiles (CC_BC_). There is an increase in concordance rates by 11.8% using GPT4 and a decrease by −17.6% between for the two GPT3.5 versions.

## Discussion

In a novel research field that still lacks methodological best practices, this work presents an early feasibility study that uses a structured approach for comparing different publicly accessible LLM for complex decision-making in a simulated environment in breast cancer care. Based on the definition provided by the FDA (United States Food and Drug Administration), an EFS represents a preliminary clinical assessment of a technological application early in its development [26]. This study type involves examining a small group of cases to assess a new technological application, focusing on its initial safety for clinical use and its functional performance. The objective of this evaluation is to gather insights that could inform potential modifications to the application before initiating larger preclinical and clinical trials. EFS build an essential step in the evidence generation process, allowing to test innovative technologies and accompany these into a healthcare setting that could bring value to patients. In the European Union, there is neither a common standardized definition of EFS nor a regulatory framework on how such studies should be methodologically designed [27]. Due to the increasing importance of evaluating technological applications for their use in the medical sector, the Europe-wide project “Harmonized Approach to Early Feasibility Studies for Medical Devices in the European Union” (HEU-EFS) was launched in October 2023 [27]. It aims to develop a validated standardized approach for EFS in the European Union to provide early insights into technology evidence. In reference to the recommendations of the FDA and the initial results and objectives of HEU-EFS, the present study was conducted to guide adaptations of LLM technology and the scientific approach to it in the context of breast cancer care.

### Principal findings

To our knowledge, this is the first dedicated early feasibility study (EFS) in breast cancer care that investigates different publicly available LLM and illustrates how they have advanced over a short time with access to more data or successive technological upgrade. It highlights a growing alignment for the GPT algorithm with complex decision-making processes in treating breast cancer, with GPT4 providing the highest concordance with the current gold standard of a multidisciplinary tumor board. This improvement appears to be primarily linked to the upgrade from GPT3.5 to GPT4 in the underlying technology. A comparison with Llama2 and Bard underscored GPT4’s superior algorithm accuracy. Furthermore, the findings support recent scientific critique of a prevailing challenge of LLM consistency over time by illustrating a declining accuracy of GPT3.5 within a six-month time period despite updated and enlarged data access, underlining the necessity for ongoing scientific monitoring of LLM [25]. These findings are important as they expand upon previous research, comparing the concordance of various LLM in managing breast cancer scenarios and monitoring advancements in accuracy over time and through continuous updates. Against the background of prior work, the results can contribute to the methodological and technological development of LLM application in breast cancer care.

### Comparison to prior work

Previous analyses pointed toward the potential of LLM in providing clinical decision support for professional users, offering medical knowledge for different specialties throughout the entire clinical process [28]. In breast cancer care, few studies have explored LLM areas of use.

Rao and colleagues showed the promising use of GPT3.5 in radiologic evaluations and screening, proving its value in mammographic imaging [29]. Additionally, Haver et al. illustrated the chatbot’s capability in providing patient education on breast cancer prevention and screening [30]. Moreover, Choi et al. demonstrated the efficiency of using tailored prompts for LLM in extracting clinical insights from pathology and ultrasound reports in extensive breast cancer medical records [31]. The quality of AI-generated abstracts has reached a level of medical appropriateness that leaves experts to find it challenging to distinguish them from specialist-written content in a blinded review process [32].

With regard to tumor board decision-making, Lukac et al. and Sorin et al. retrospectively compared the answers of GPT3.5 (version September 2021) to the past treatment recommendations of a single tumor board [22, 23]. The latter research represents initial explorations of this technology, rather than definitive benchmarks for evaluating the capabilities of ChatGPT3.5. Their experiments only included the LLM ChatGPT3.5, involved a constrained and unstructured collection of patient profiles with restricted health data, and they utilized a short and limited prompting strategy. Additionally, their assessments were based on a self-developed scoring system. Notably, the studies omitted genetic testing for most cases, which is a crucial factor in the characterization of breast cancer. Both preliminary assessments inferred from their findings that the advice given by language model-based systems could align with that of a tumor board, but refrain from definitive statements about the specific performance level of LLM in their conclusions. Our research builds on the findings of Lukac et al. and Sorin et al. and seeks to extend them in a systematic manner [22, 23]. Therefore, we confirmed GPT3.5’s potential for managing high complexity case by employing a standardized prompting model and using comprehensive health data profiles as described in the methodology [24]. This subsequent EFS provides further insights by comparing different LLM versions and monitoring development over time, with access to more data and technological upgrade. It matches a generic observation by Eriksen et al. of superior performance by GPT4 for diagnosing complex clinical cases and confirms this finding in the field of breast cancer care [33]. Furthermore, it confirms the most recently raised critique of LLM regarding a persisting challenge in answer consistency in the field of breast cancer treatment [25] This relates to the deterioration in GPT3.5’s accuracy over the observation period. It points toward the possible issue, that an extension of data access with uncontrolled sources used for decision-making does not necessarily lead to an improvement in LLM accuracy but could lead to confusion in the models.

### Limitations and implications for methodological and technological development of LLM application in breast cancer care

By monitoring the evolution of LLM, this study shows that especially the update to the GPT4 algorithm enables an increasing alignment with the recommendations of the MTB. It indicates that technological applicability rapidly develops toward technological maturity to provide clinical decision support, even for complex decision-making in breast cancer care. Nevertheless, at present, the study also underlines that a clinical use of LLM is not yet feasible. Several unresolved regulatory hurdles and missing evidence on the peculiarities of clinical application should forbid their current use in clinical care. The current level of evidence regarding the use of LLM in breast cancer therapy leaves crucial questions unanswered, which can also be derived this study.

The initially chosen prompting model only required the LLM to indicate whether chemotherapy should be given or not. However, the recommendation of multi-gene assays to assess disease prognosis and predict chemotherapy benefit in patients was not queried. Due to the increasing use of such tests and the associated increasing clinical relevance, future prompting models should include a query relating to the need for multi-gene assays to assess the chemotherapy necessity. This finding underscores the methodological need to develop sophisticated prompting models that should be tailored to the specifics of the oncologic entity being investigated in order to improve the consistency in LLM answering.

Furthermore, the study uses the recommendations by a single MTB as gold standard for comparing concordance in LLM decision-making. Large-scale observational studies, conducted by several international study groups, have revealed notable disparities in breast cancer treatment choices and outcomes [34, 35]. There is often considerable scope for decision-making on available treatment options, such as varying intensities of chemotherapy regimens, which reflects the diversity in national standards and respective guidelines. This issue also explains the rather moderate results for DCIS profiles in this study. The LLM have consistently recommended endocrine therapy, as, for example, suggested in a meta-analysis by Yan et al. from 2020 [36]. In contrast, the MTB in the study decided against endocrine therapy in the DCIS cases, a decision that was taken in interdisciplinary discourse in the MTB and within the decision-making scope of the German guidelines. However, as a dedicated EFS with a small group of 20 cases, no conclusions should be drawn regarding the LLM accuracy for different cancer subtypes stages of the disease, i.e., precancerous or advanced metastasized illness, and treatment options. Hence, in order to ensure the evidence-based and safe use of LLM in breast cancer care, these open questions must be adequately addressed by further research. Subsequent studies should incorporate larger study populations and multicenter study designs to expand findings from a preclinical simulation environment into clinical care.

At a technological level, a lack of control over the sources used for decision-making and a lack of security in the processing of health data have so far prevented the use of LLM in clinical care. The deterioration in GPT3.5’s accuracy over the observation period, which appears to be connected to the extension of data access, underlines how uncontrolled and enlarged input of sources can contribute to confusion in the models. It remains unclear which sources the open LLM use for decision-making, a problem that can also be seen in the moderate DCIS results, as it cannot be derived from the LLM answering which evidence is used by the LLM to recommend endocrine therapy. In alignment with the Explainable AI approach, the technological application should offer the possibility of gaining control over the sources used for decision-making while ensuring security in the processing of personal health data, i.e., by limiting it to local servers.

### Opportunities for breast cancer care

Considering the findings of the national survey conducted by the Commission Digital Medicine of the German Association of Gynecology and Obstetrics (DGGG), 61.4% of specialists either agree of strongly agree that intelligent algorithms will support clinicians to treat patients and the majority support the perception that this will improve patient care (65.1% agree of strongly agree) and help to reduce increasing workload (78.4% agree of strongly agree) [12]. These concerns are accompanied by the aforementioned, intensified care complexity due to the rapid increase in evidence-based knowledge and case load in gynecological oncology [4, 5]. In this perspective, easily accessible and user-friendly publicly available LLM may provide a prospective solution in breaking down prevailing barriers [37]. As presented in this study, a clinical use of LLM is not yet feasible. Nevertheless, the controlled and evidence-based adaptation of LLM , i.e., the optimization of prompting techniques or enabling data input control and secure data processing, offers potential that LLM could bring value to patients in clinical breast cancer care.

### Conclusion

This early feasibility study demonstrates that publicly available LLM, especially GPT4, increasingly align with the decision-making of a multidisciplinary tumor board and confirms decision consistency to remain a major issue for the application of LLM in breast cancer care. The findings underline that clinical use of LLM is not yet feasible. Nevertheless, the study gathers insights that could inform potential modifications to the LLM application. Methodological advancement, i.e., the optimization of prompting techniques, and technological development, i.e., enabling data input control and secure data processing, are necessary in the preparation of large-scale and multi-centric studies. These will subsequently provide further essential evidence on the safe and reliable application of LLM in breast cancer care to maximize benefits for providers and patients alike.

## Data Availability

Datasets generated and analyzed during the current study are available from the corresponding author on reasonable request.
